# Metabolomic Analysis of the Responses of Bryophyte *Tortella tortuosa* (Hedw.) Limpr. to Cadmium (Cd) Stress

**DOI:** 10.3390/ijms26072856

**Published:** 2025-03-21

**Authors:** Yongqi Zhu, Dongmei Lin, Qiuge Li, Mengjie An, Jie Lv

**Affiliations:** Key Laboratory of Biological Resources and Genetic Engineering of Xinjiang, College of Life Science and Technology, Xinjiang University, Urumqi 830046, China; yongqizhu@xju.edu.cn (Y.Z.);

**Keywords:** bryophyte, heavy metal stress, differentially accumulated metabolites, metabolic pathways

## Abstract

In recent years, there have been many studies on the response of plants to heavy metal stress, but the metabolic changes in bryophytes, pioneer plants quickly responding to environmental changes, under exogenous cadmium (Cd) stress have yet to be explored. In this indoor experiment, the responses in the metabolome of bryophyte *Tortella tortuosa* (Hedw.) Limpr. to different Cd exposure levels (0 (CK), 5 (T1), and 10 (T2) mg·L^−1^) were analyzed. The results showed that the number of differentially accumulated metabolites (DAMs) secreted by *T. tortuosa* increased with the increase in the Cd concentration, and the biosynthesis of cofactors, D-Amino acid metabolism, Arginine biosynthesis, ATP-binding cassette transporters (ABC transporters), and biosynthesis of alkaloids derived from shikimate pathway were the main pathways enriched by DAMs. The relative abundances of malic acid, N-Formylkynurenine, L-Glutamine, L-Histidine, LL-2,6-Diaminopimelic acid, and fusaric acid in the T2 treatment increased by 16.06%, 62.51%, 14.51%, 11.92%, 21.37%, and 35.79%, respectively (*p* < 0.05), compared with those of the CK, and the correlation analysis results showed that the above DAMs were closely related to the changes in plant antioxidant enzyme activity and Cd concentration. These results indicate that the secretion of amino acid (N-Formylkynurenine, L-Histidine) and organic acids (isocitric acid, LL-2,6-Diaminopimelic acid, malic acid) through the metabolic pathways, including biosynthesis of amino acids, biosynthesis of cofactors, glyoxylate and dicarboxylate metabolism, and ABC transporters, is the metabolic mechanism of *T. tortuosa* to resist exogenous Cd stress. This study will provide a reference for the monitoring and remediation of heavy metal pollution.

## 1. Introduction

Heavy metal ions enter terrestrial ecosystems through various pathways, such as rock weathering, application of chemical fertilizers and pesticides, waste incineration, mining, and vehicle emissions [1,2]. This ultimately threatens human health through the plants’ absorption and accumulation of heavy metals. Metal elements such as manganese (Mn), zinc (Zn), copper (Cu), iron (Fe), cobalt (Co), nickel (Ni), selenium (Se), and molybdenum (Mo) are essential components of various biological processes in plants [3,4]. However, non-essential heavy metals, including arsenic (As), chromium (Cr), cadmium (Cd), mercury (Hg), silver (Ag), and lead (Pb), exert toxic effects and disrupt normal physiological activities of plants by competing for protein-binding sites, causing plants to wilt and even die [5,6].

Bryophytes do not have cuticula and are short in height, but its body surface area is large. Therefore, bryophytes have a good electrostatic adsorption performance, and its special plant structure and physiological characteristics make it respond very quickly to the changes in heavy metal ions in the environment [7,8]. Heavy metals can damage the intracellular structure of bryophytes, resulting in separation between cytoplasm and wall and cell wall thickening. Heavy metals can also lead to the accumulation of methylglyoxal in the cytoplasm of plants, which aggravates the damage. Hossain et al. [9] found that heavy metal stress damaged many enzymes that were essential for plant metabolism (superoxide dismutase, glutathione reductase, peroxidase, etc.), leading to protein denaturation, loss of function of cell membranes, reduced photosynthesis and respiration, as well as imbalances of ROS (reactive oxygen species). However, the cytoplasmic membrane of plants can make it impossible for heavy metal ions to penetrate the organelles and hinder the adverse effects of heavy metals on the plants. When bryophytes are exposed to heavy metals, the metabolic balance of free radicals in cells is disrupted, including the formation of reactive oxygen species (ROS) (superoxide anion (O_2_^·−^), hydroxyl radical (·OH), and hydrogen peroxide (H_2_O_2_)). This leads to oxidative stress in plants, damages cell membranes and DNA strands, and even triggers cell death.

Metabolomics can reflect the subtle changes in the metabolism of plants under environmental stress conditions. Fu et al. [10] found that the contents of organic acids (oxalic acid, tartaric acid, malic acid, citric acid, succinic acid, malonic acid, and acetic acid) and amino acids (lysine, glycine, alanine, methionine, glutamic acid, and histidine) in rice root exudates increased under Cd stress, with the concentrations of tartaric acid and histidine being the highest. Fu et al. [10] also found that exogenous Cd stress seriously affected the secondary metabolism and purine, amino acid, glyceride, and carbon metabolism pathways of rice. Exogenous small-molecule compounds could enhance the uptake of Cd by plants and affect the methylation status [11,12,13]. However, E. Ali et al. [14] found that the tocopherols secreted by rapeseed roots could prevent the uptake of Cd by rapeseed roots and limit its transport to aboveground tissues. Under Cd stress, increasing the concentration of in vivo α-tocopherol could help reduce the negative impact of Cd ions on *Arabidopsis thaliana* [15], while metabolites such as glutathione (GSH) and tocopherol in bryophytes could alleviate Hg stress by reducing mercury concentration and redox damage [16]. Under mild Cd treatment, the rapid expression of GST in *Leptodictyum riparium* (Hedw.) Warnst could be induced to synthesize a large amount of glutathione, which helped alleviate Cd stress by detoxification and chelation [8,16,17,18].

In recent years, metabolomics has been widely used in the studies of animal pathology, plants’ stress responses, food nutrition, soil, etc., among which the metabolomics research on plant stress response is mostly applied to widely planted crops, some higher plants, and model plants. However, the metabolomic studies on bryophytes under heavy metal stress are few. Previous studies on bryophyte responses to heavy metal stress have primarily focused on their utility as bioindicators for heavy metal pollution [8] and antioxidative enzyme dynamics (e.g., peroxidase activity modulation) [19]. Although transcriptomic insights into stress-responsive genes have been preliminarily explored [20], the metabolomic basis underlying their adaptation mechanisms remains poorly characterized. Especially, currently, the metabolic mechanism of *T. tortuosa* in response to Cd stress is still unclear. Based on the existing studies, the following hypotheses were proposed: (1) the types and abundance of metabolites in *T. tortuosa* might vary with Cd concentrations; and (2) *T. tortuosa* might resist Cd stress by regulating the abundance of key metabolites and the corresponding metabolic pathways. To test the hypotheses, in this indoor experiment, the responses in the metabolome of *T. tortuosa* to different Cd exposure levels (0 (CK), 5 (T1), and 10 (T2) mg·L^−1^) were analyzed. The objectives of this study were to determine (1) the types and abundance of differentially abundant metabolites (DAMs) of *T. tortuosa* in response to Cd stress, (2) the correlation of key DAMs with Cd content and physiological indicators, and (3) the key metabolic pathways of *T. tortuosa* in response to Cd stress. This study systematically investigated cadmium (Cd) absorption capacity in bryophytes across a gradient of environmentally relevant Cd concentrations (0, 5, 10 mg/L) and delineated their metabolic reprogramming under Cd stress. Our findings bridge this critical knowledge gap by identifying dose-dependent metabolic shifts, including significant upregulation of amino acids (N-formylkynurenine, L-histidine) and organic acids (isocitric acid, malic acid), which can optimize bryophyte-based Cd remediation strategies through targeted metabolic engineering.

## 2. Results

### 2.1. Data Preprocessing

**Figure 1:**
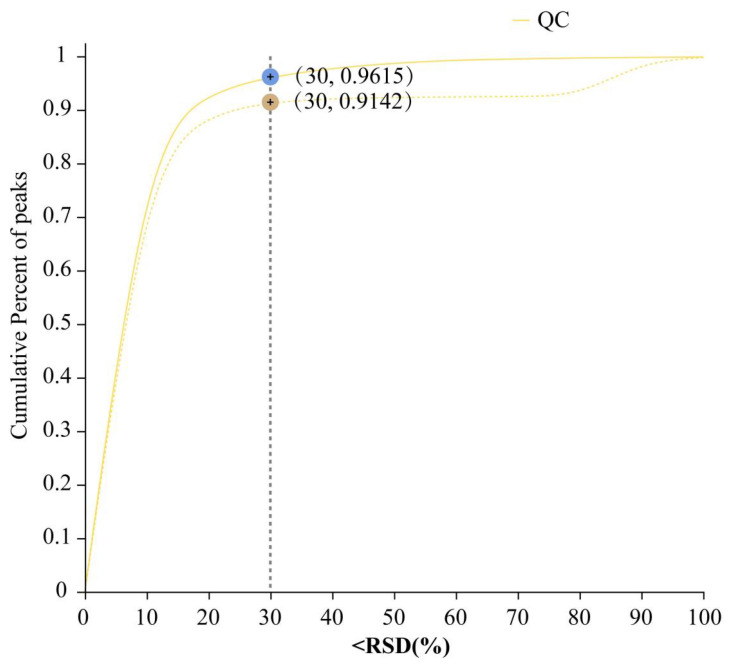
Validation of the raw data. Relative standard deviation (S/Mean) (%) value, i.e., the standard deviation/mean, cumulative percent of peaks. Pre_QC (dashed line): Post-quality control data distribution, reflecting variability improvement. Raw_QC (solid line): Original data distribution.

For the whole data, the relative standard deviation (S/Mean) was smaller than 0.3, and the cumulative percent of peaks was greater than 70% (Figure 1). Therefore, the data of the metabolites obtained were valid and could be used for subsequent differential analysis.

### 2.2. Expression of DAMs

**Figure 2:**
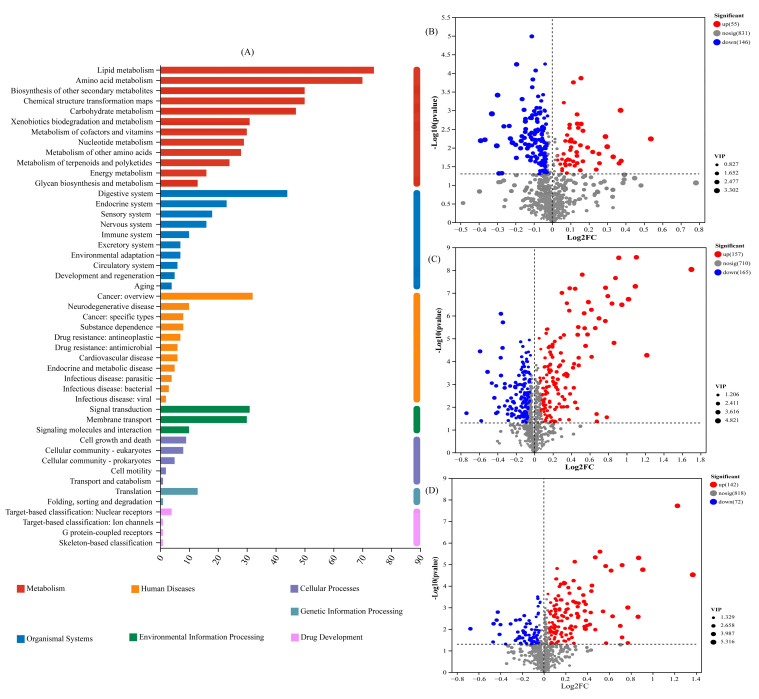
Statistics of key pathways and expression of differentially abundant metabolites (DAMs): (**A**) KEGG pathways enriched by DAMs; (**B**) statistics of up- and down-regulated metabolites in T1 vs. CK; (**C**) statistics of up- and down-regulated metabolites in T2 vs. CK; (**D**) statistics of up- and down-regulated metabolites in T1 vs. T2. The x-axis represents the fold-change of the compared groups. The y-axis indicates the significance of differential expression. Gray spots indicate no significant difference, whereas red and blue spots indicate up-and down-regulated unigenes, respectively (*p* value < 0.005 and |log2 FC| > 1).

The number of DAMs detected in the metabolism pathway (first level) was the largest, with 74 and 70 DAMs in the lipid metabolism and amino acid metabolism pathways (second level), respectively (Figure 2A). There were 55 up-regulated DAMs and 146 down-regulated DAMs in the T1 treatment compared with those in the CK (Figure 2B). There were 157 up-regulated DAMs and 165 down-regulated DAMs in the T2 treatment compared with those in the CK (Figure 2C). There were 142 up-regulated DAMs and 72 down-regulated DAMs in the T1 treatment compared with those in the T2 treatment (Figure 2D).

### 2.3. Pathway Enrichment Analysis

**Figure 3:**
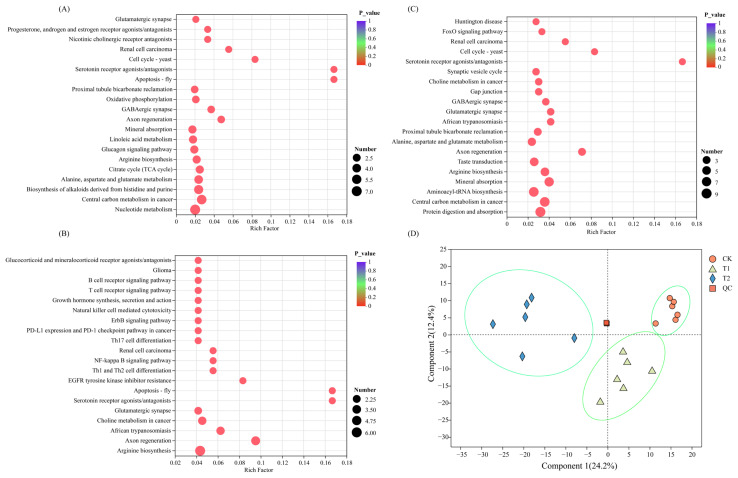
Statistics of DAM relative expression in the CK (**A**), T1 (**B**), and T2 (**C**) treatment and PLSDA analysis (**D**). The abscissa is the enrichment rate, and the calculation method is num_in_study/num_in_pop; the ordinate is the KEGG pathways. The size of the bubble represents the amount of DAMs enriched in the pathway; the larger bubble, the more the DAMs enriched in the pathway.

The DAMs-enriched pathways were different for different pairwise comparison groups. The DAMs of T1 vs. CK and T2 vs. CK were mainly enriched in the biosynthesis of cofactors, D-Amino acid metabolism, Arginine biosynthesis, ABC transporters, and biosynthesis of alkaloids derived from Shikimate pathways (Figure 3A,B). The DAMs of T1 vs. T2 were mainly enriched in the ABC transporters, amino acid metabolism, and Aminoacyl-tRNA biosynthesis (Figure 3C).

### 2.4. Visual Analysis of DAMs-Enriched Metabolic Pathways

**Figure 4:**
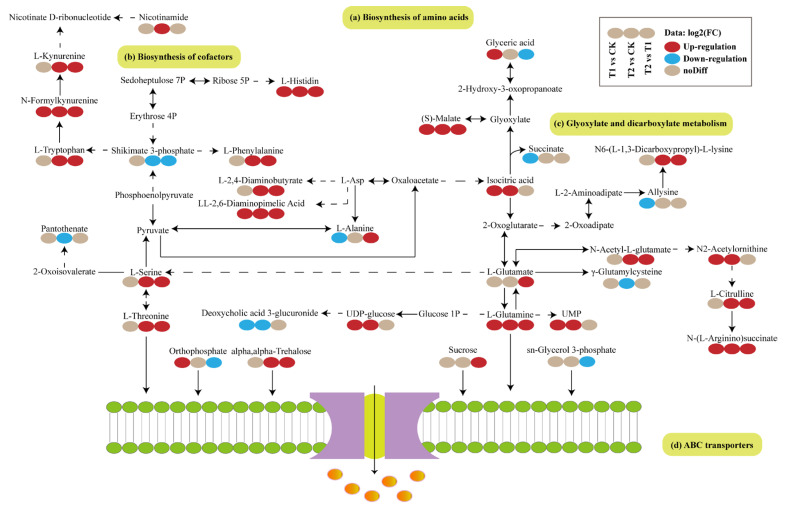
Visual analysis of the metabolic pathways in *Tortella tortuosa* in response to exogenous Cd stress based on the relative abundances of DAMs. Red indicates up-regulation, and blue indicates down-regulation. Solid line represents direct relationship, and dotted line indicates indirect relationship. Solid lines: Direct metabolic pathways. Dashed lines: Indirect interactions.

The DAMs of T1 vs. CK, T2 vs. CK, and T1 vs. T2 were extracted and integrated to obtain the metabolic pathways of *T. tortuosa* in response to exogenous Cd stress, including biosynthesis of amino acids, biosynthesis of cofactors, glyoxylate and dicarboxylate metabolism, and ABC transporters pathways (Figure 4). N-Formylkynurenine, L-Histidin, (S)-Malate, Isocitric acid, LL-2,6-diaminopimelic acid, N2-Acetylornithine, L-Glutamine, and N-(L-Arginino)succinate in the T1 and T2 treatments were up-regulated (*p* < 0.05), and Deoxycholic acid 3-glucuronide was down-regulated (*p* < 0.05), compared with those in the CK. Shikimate 3-phosphate, Glyceric acid, Orthophosphate, and sn-Glycerol 3-phosphate in the T1 treatment were down-regulated compared with those in the T2 treatment (*p* < 0.05).

### 2.5. Differential Metabolite Abundance Profiles

**Figure 5:**
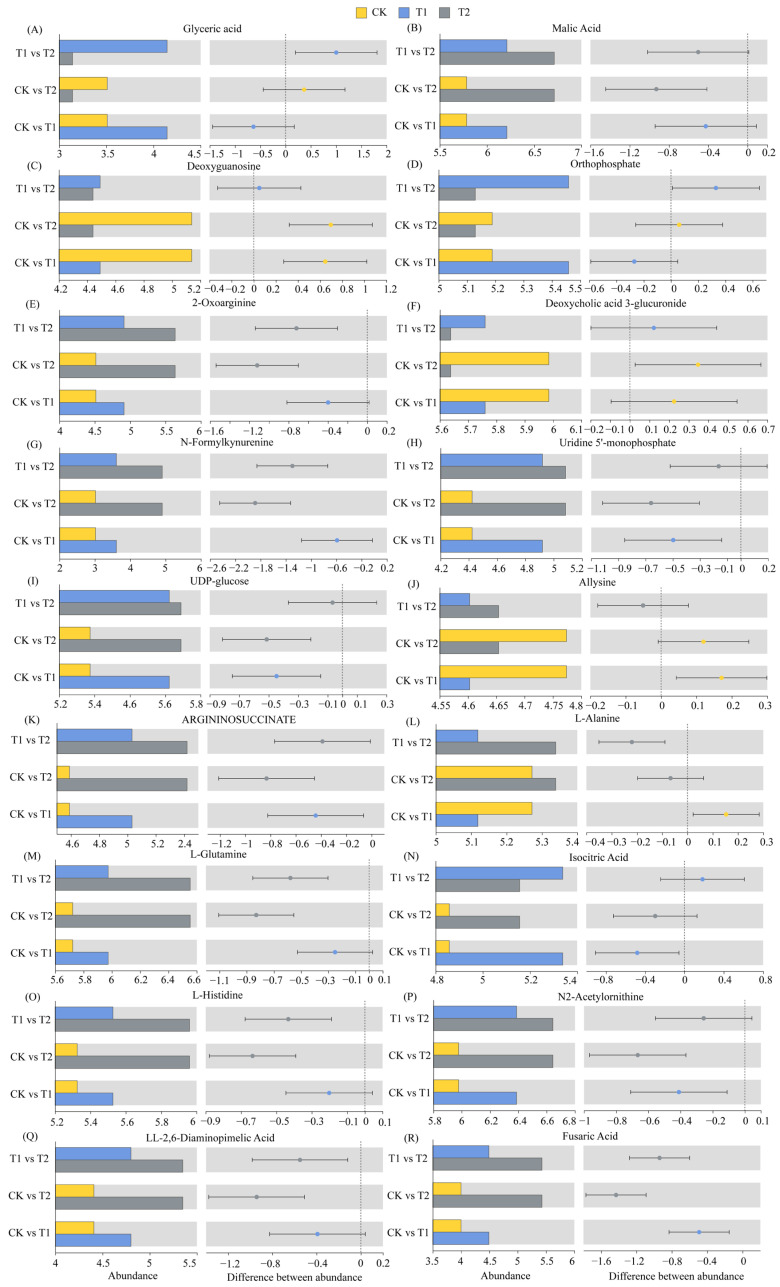
Relative abundances of differentially abundant metabolites.

The relative abundances of glyceric acid, malic acid, 2-Oxoarginine, N-Formylkynurenine, Uridine 5′-monophosphate, UDP-glucose, ARGININOSUCCINATE, L-Glutamine, isocitric acid, L-Histidine, N2-Acetylornithine, LL-2,6-Diaminopimelic acid, and fusaric acid in the T1 treatment increased by 18.11%, 7.36%, 8.84%, 19.54%, 11.24%, 8.32%, 9.65%, 4.41%, 9.91%, 3.79%, 6.86%, 8.89%, and 12.31%, respectively (*p* < 0.05), compared with those in the CK treatment (Figure 5A,B,E,G–I,K,M–R). The relative abundances of malic acid, 2-Oxoarginine, N-Formylkynurenine, Uridine 5′-monophosphate, UDP-glucose, ARGININOSUCCINATE, L-Glutamine, isocitric acid, L-Histidine, N2-Acetylornithine, LL-2,6-Diaminopimelic acid, and fusaric acid in the T2 treatment increased by 16.06%, 24.81%, 62.51%, 14.92%, 9.56%, 18.13%, 14.51%, 6.13%, 11.92%, 11.14%, 21.37%, and 35.79%, respectively (*p* < 0.05), compared with those in the CK treatment (Figure 5B,E,G–I,K,M–R). The relative abundances of Deoxyguanosine, Deoxycholic acid 3-glucuronide, and Allysine in the T1 treatment decreased by 12.53%, 3.76%, and 3.58%, respectively (*p* < 0.05) (Figure 5C,F,J), and those of Deoxyguanosine, Deoxycholic acid 3-glucuronide, Allysine, and L-Alanine in the T2 treatment decreased by 13.57%, 5.81%, 2.51%, and 2.91%, respectively (*p* < 0.05), compared with those in the CK treatment (Figure 5C,F,J,L).

### 2.6. Correlation Analysis Between DAMs and T. tortuosa Physiological Characteristics and Cd Content

**Figure 6:**
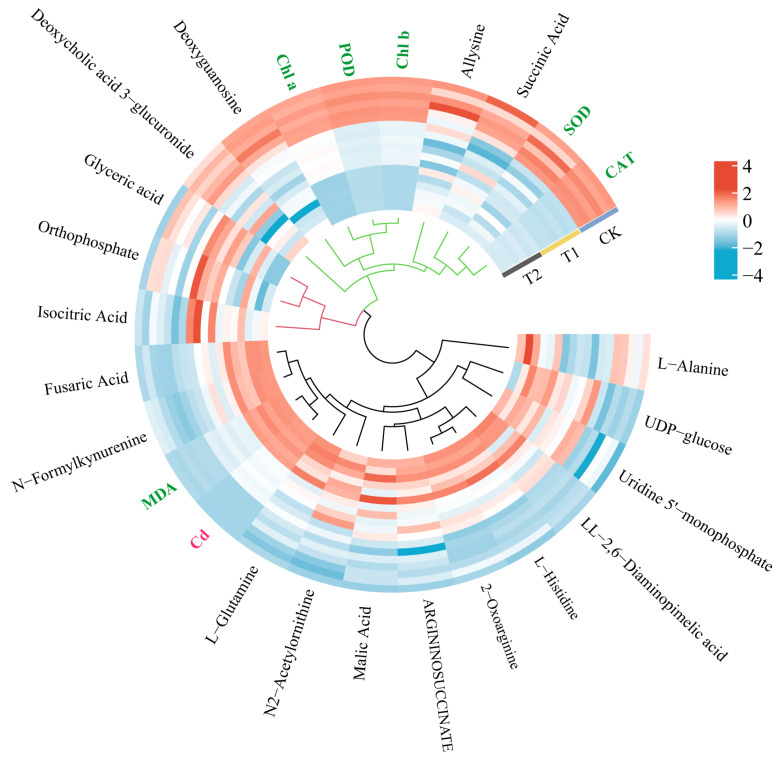
Heat map analysis of DAMs and physiological characteristics. Note: The hierarchical clustering of indicators based on correlation analysis revealed three distinct clusters: Cluster 1 (black): 14 indicators significantly correlated with cadmium content (Cd); Cluster 2 (green): 9 indicators strongly linked to physiological parameters; Cluster 3 (red): 3 independent metabolites showing low correlation with physiological parameters. (Color codes: Cd [red]; physiological parameters [green]; metabolites [black]).

There were three main clusters (Figure 6). The largest cluster included L-Alanine, L-Glutamine, L-Histidine, N2-Acetylornithine, LL-2,6-Diaminopimelic acid, ARGININOSUCCINATE, UDP-glucose, and Uridine 5′-monophosphate, N-Formylkynurenine, 2-oxoarginine, malic acid, fusaric acid, Cd content, and MDA content, among which the key metabolites, fusaric acid and N-Formylkynurenine, were closely related to the changes in Cd content and MDA content in *T. tortuosa.* The heat map (Figure 6) showed that the relative abundances of fusaric acid, N-Formylkynurenine, Cd content, and MDA content in the T1 and T2 treatments increased compared with those in the CK. Therefore, the relative abundances of fusaric acid and N-Formylkynurenine increased with the increase in Cd and MDA contents in *T. tortuosa*. The second cluster included Allysine, Deoxycholic acid 3-glucuronide, Deoxyguanosine, succinic acid, SOD activity, CAT activity, POD activity, Chl a content, and Chl b content, among which the key metabolites, Allysine and succinic acid, were closely related to the changes in SOD and CAT activities in *T. tortuosa*, and Deoxyguanosine was closely related to changes in POD activity and Chl a and Chl b contents. The heat map (Figure 6) showed that the relative abundances of Allysine, succinic acid, Deoxyguanosine, SOD activity, CAT activity, POD activity, and Chl a and Chl b contents in the T2 treatment decreased compared with those in the CK. Therefore, the relative abundances of Allysine and succinic acid decreased with the decrease in SOD and CAT activities in *T. tortuosa*, and Deoxyguanosine decreased with the decrease in POD activity and Chl a and Chl b content. The third cluster included isocitric acid, Orthophosphate, and glyceric acid, which were not closely related to physiological indicators.

## 3. Discussion

Bryophytes exhibit strong adsorption capacity for heavy metals, owing to their multiple branches, large surface area, and the lack of wax layer. Bryophytes can absorb heavy metals from both dorsal and ventral sides. In this study, the adsorption of Cd by *T. tortuosa* increased with the increase in exogenous Cd concentration (Table S1). However, previous studies have found that bryophytes have some differences in their capacity to absorb heavy metals, due to the differences in the water-holding capacity of different bryophytes [21,22,23]. Salemaa et al. [24] found that the adsorption capacity of *Pohlia nutans* Lindb. for heavy metals was higher than that of other bryophytes, and it was significantly affected by the growth substrate, bryophyte species, growth substrate pH and temperature, and habitat conditions. In this study, the response of chlorophyll content in bryophytes to heavy metals was very significant. This indicates that bryophytes are suitable as bioindicators for monitoring heavy metal pollution. Lou et al. [25] observed that when the Pb^2+^ concentration was in the range of 0–400 mg/L, the POD activity of bryophytes *Haplocladium microphyllum* and *Brachythecium procumbens* gradually increased with the increase in heavy metal concentration; however, in *Physcomitrella patens*, with the Pb^2+^ concentration of 200 mg/L serving as a pivotal point, the POD activity exhibited a trend of initially increasing and subsequently decreasing. Therefore, it is suggested that the POD activity and MDA content of bryophytes could be used as auxiliary physiological indicators for monitoring environmental heavy metal pollution. Heavy metal ions affect plant growth by interfering with the metabolite biosynthesis and changing the rhizosphere microenvironment [2]. In this study, a total of 476 DAMs were selected, among which amino acids, carboxylic acids, oleic acids, and fatty acids were the dominant ones (Figure 2). Studies have shown that the relative abundances of most alkaloids, lipids, flavonoids, and organic acids increase under different concentrations of Cd conditions, indicating that the above metabolites are the key substances for plants to resist exogenous Cd stress [26,27,28,29]. For example, Sun et al. [30] identified 33 *Sedum erythrostictum* metabolites that showed significant difference between Cd stress treatment and CK treatment, including amino acids, lipids, organic acids, and polyols. Guo et al. [1] found that under salt stress, the releases of organic acids (including oxalic acid, malic acid, fumaric acid, piperidic acid, glyceric acid, threonic acid, and 3-hydroxybutyric acid) in the rhizosphere of edible *Amaranthus tricolor* L. cultivars increased compared with those in the CK, which significantly promoted the migration of soil Cd to plants. Xiao et al. [31] found that plant root exudates (lipids, terpenoids, flavonoids, and organic acids) could reduce Cr toxicity to plants through different mechanisms of action, thereby enhancing plant tolerance to Cr stress [32,33].

In this study, biosynthesis of amino acids, biosynthesis of cofactors, glyoxylate and dicarboxylate metabolism, and ABC transporters were the main metabolic pathways significantly enriched by DAMs under Cd treatments (Figure 4). Therefore, the above metabolic pathways are the mechanisms of *T. tortuosa* in response to exogenous Cd stress. The DAMs involved in the amino acid metabolism are very important in the resistance of plants to external stress [34,35]. Studies have shown that the free amino acids of non-protein origin in the amino acid metabolic pathway, ubiquitous in prokaryotes and eukaryotes, for regulating carbon/nitrogen ratio (C/N ratio), pH, and osmolytes in plants [36,37,38]. The metabolism of fatty acids (FAs) plays a crucial role in plants’ stress resistance mechanism [39], is a precursor of the signal synthesis of plant hormones jasmonic acid (JA), and also interacts with various plant hormones (salicylic acid (SA), jasmonic acid (JA), and abscisic acid) [40,41,42]. ABC transporters not only play an important role in zebrafish coping with heavy metal stress but also widely exist in plants to resist external stress [43]. ABC transporters transport various substrates through extracellular and intracellular transmembrane and participate in the absorption of nutrients and the efflux of toxic substances, such as amino acids, nucleotides, carbohydrates, lipids (e.g., cholesterol, steroids), vitamins, peptides, glutathione conjugates, heavy metal chelates, and exogenous substances (e.g., antibiotics, herbicides) [44]. In this study, Orthophosphate, L-Glutamine, L-Alanine, L-Serine, L-Threonine, L-Histidine, L-Glutamic Acid, Deoxyguanosine, Uridine, Sucrose, L-Phenylalanine, Trehalose, and L-Glutamate were significantly enriched in the ABC transporters and increased under T1 and T2 treatments, compared with those in the CK (Table S2). This indicates that these substances showed increased transport of substrates and endogenous toxins under heavy metal stress. It has been reported that amino acids are the precursors and main components of protein synthesis and play an important role in plant metabolism and development, and the synthesis and accumulation of amino acids are the resistance mechanism of plants under stress [36,45]. In addition to playing a role in carbon and energy storage during limited growth and photosynthesis under stress, amino acids also play an active role in maintaining stable enzyme activity and intact lipid membranes [46,47,48]. Amino acids also play an important role in metal binding, signaling, and antioxidant defense in plants under heavy metal stresses [49]. In addition to amino acids, organic acid compounds, such as citric acid and malic acid, are essential in the tricarboxylic acid (TCA) cycle and play an important role in resisting exogenous Cd toxicity [50,51]. Xu et al. [52] showed that under Cd stress, the relative abundances of organic acid compounds isocitric acid, malic acid, glyceric acid, 3-hydroxybutyric, and fumaric acids in *Elodea canadensis* Michx were 0.81, 2.48, 1.95, 40.2, and 4.10 times that of the control, respectively. This study obtained similar results (Figure 5). Contrary to the typical response in vascular plants under heavy metal stress—where accelerated TCA cycle activity leads to dose-dependent accumulation of both citrate and isocitrate, with citrate exhibiting higher abundance [53]—our study revealed a distinct pattern in *T. tortuosa*: isocitrate levels surpassed citrate under Cd exposure. We propose two possibilities to explain this divergence: (1) if sampling occurred during a metabolically active phase (e.g., peak energy demand), citrate might have been preferentially consumed via the TCA cycle, while isocitrate accumulation could result from rate-limiting downstream reactions mediated by isocitrate dehydrogenase; (2) this study employed an untargeted liquid chromatography–mass spectrometry (LC-MS) metabolomics approach. Under the electrospray ionization conditions applied (positive ion mode, 3.5 kV), the ionization efficiency of isocitrate may have exceeded that of citrate, potentially introducing differential detection sensitivity between these two metabolites. This work highlights the necessity of species-specific mechanistic frameworks. However, current metabolomic studies addressing heavy metal stress responses in bryophytes remain in their infancy. Future investigations should aim to resolve these mechanistic uncertainties and propose novel perspectives on their unique detoxification strategies.

In this study, the key metabolites fusaric Acid and N-Formylkynurenine were closely related to the Cd content in *T. tortuosa* (Figure 6), and their relative abundances significantly increased under exogenous Cd stress compared with those in the CK (Figure 5). Fusaric acid is one of the most harmful phytotoxins produced in various plant–pathogen interactions [54]. The ROS induced by fusaric acid affects the enzymatic and non-enzymatic antioxidant systems regulated by plant hormones and causes adverse effects, including mitochondrial dysfunction and lipid peroxidation, which ultimately inhibit plant growth and development and reduced crop yields [54,55]. In this study, the relative abundance of allysine, succinic acid, and deoxyguanosine were positively correlated with SOD activity, CAT activity, POD activity, and Chl a and Chl b content in T2 treatment (Figure 6). Song et al. [56] and Duan et al. [57] showed that the application of succinic acid increased the POD activity and chlorophyll content of *Larix olgensis* roots, stems, and leaves under Cd and Pb treatments and reduced the oxidative damage and MDA content compared with the CK. This study obtained similar results. This indicates that the secretion of succinic acid by *T. tortuosa* helps to resist oxidative damage caused by Cd stress (Figure 6, Table S2) [58]. In other words, the secretion of amino acids and organic acids helps *T. tortuosa* to resist external abiotic stresses. This provides reference for the remediation of heavy metal pollution.

## 4. Materials and Methods

### 4.1. Study Area

The plant-sampling site was located in the Tianshan Mountains in the south of Urumqi, Xinjiang, China (43°07′09″–43°27′42″ N, 87°04′30″–87°29′15″ E), spanning 60 km from south to north, with a total area of 120 km^2^. This region has a temperate continental climate, with 40% of the annual precipitation concentrated in June–August. The average annual precipitation is 456 mm. Forests are mainly distributed in the northern slope in the altitude range of 1500 m–2800 m. More than 90% of the trees in the forests are *Piceas chrenkiana*.

### 4.2. Experimental Design

The pretest results showed that when the Cd concentration in the hydroponic solution reached 10 mg·L^−1^, *T. tortuosa* plants showed severe wilting. This experiment design included two different concentrations of Cd (T1, 5 mg·L^−1^; T2, 10 mg·L^−1^) and a control treatment (CK). Each treatment had three replicates/pots. The collected *T. tortuosa* plants (8.0 g) were rinsed with water and then placed in a transparent box containing 60 mL Knudoson C nutrient solution (prepared with 1000 mL distilled water, 1000 mg Ca(NO_3_)_2_·4H_2_O, 250 mg MgSO_4_·7H_2_O, 250 mg KH_2_PO_4_, 500 mg (NH_4_)SO_4_, 25 mg FeSO_4_·7H_2_O, and 7.5 mg MnSO_4_·4H_2_O). After that, the boxes were placed in incubator (light density: 2000 lux; light time: 16 h; humidity: 75%; temperature: 20 °C). Cadmium chloride (CdCl_2_·2.5H_2_O) and ultrapure water were used to prepare 500 mL Cd solution with a Cd concentration of 5 mg·L^−1^ and 500 mL Cd solution with a Cd concentration of 10 mg·L^−1^. The Cd solutions of different concentrations were evenly sprayed on the surface of *T. tortuosa* for 12 times, and the plants in the CK were sprayed with ultrapure water. The growth status of *T. tortuosa* was checked and recorded during Cd solution spraying. To minimize algae growth in the Knudoson C nutrient solution, the nutrient solution was diluted 10 times to reduce the contents of P and N to 33% [59]. A portion of fresh plant samples were collected after 50 days of incubation and stored in a −80 °C freezer for metabolomic assays, and the remaining samples were used to determine chlorophyll content, antioxidant enzyme activity, and malondialdehyde (MDA) content.

### 4.3. Determination of Physiological Indices

Acetone (80%) was used to extract chlorophyll, followed by colorimetry at 663 nm and 646 nm. The concentrations of Chl a and Chl b were calculated according to the equations described by Shakya (2008) [60]. The activities of antioxidant enzymes, including superoxide dismutase (SOD), catalase (CAT), and peroxydase (POD), and the content of malondialdehyde (MDA) were determined by the method of Paoletti et al. [61], Cakmak and Marschner [62], and Bharwana et al. [3] (data are shown in the Supplementary Materials).

### 4.4. Metabolomic Assays

Fresh plant samples were rinsed with deionized water, segmented into 0.5 cm pieces, flash-frozen in liquid nitrogen, and stored at −80 °C as triplicate biological replicates. The samples were subsequently processed by Shanghai Meiji Biotechnology Co., Ltd. (Shanghai, China), which included metabolite profiling, data preprocessing, metabolite identification and annotation, data quality control (QC), and differential metabolic analysis. For extraction, 50 mg aliquots were homogenized in 2 mL tubes with 6 mm grinding beads using 400 μL methanol/water (4:1, *v*/*v*) containing 0.02 mg/mL L-2-chlorophenylalanine (internal standard). The homogenate was cryogenically ground (−10 °C, 50 Hz, 6 min; Wonbio-96c, Shanghai Wanbo Biotechnology, Shanghai, China), ultrasonicated (5 °C, 40 kHz, 30 min), and incubated at −20 °C for 30 min. After centrifugation (13,000× *g*, 4 °C, 15 min), supernatants were collected for LC-MS/MS analysis.

The analysis was conducted at Majorbio Bio-Pharm Technology Co., Ltd. (Shanghai, China). Chromatographic separation was performed on a Thermo Scientific™ UHPLC-Q Exactive HF-X system (ACQUITY UPLC HSS T3 column, 100 mm × 2.1 mm, 1.8 μm; Waters, Milford, MA, USA) with mobile phases: Solvent A: 0.1% formic acid in water/acetonitrile (95:5, *v*/*v*); Solvent B: 0.1% formic acid in acetonitrile/isopropanol/water (47.5:47.5:5, *v*/*v*/*v*). Parameters: flow rate 0.40 mL/min, column temperature 40 °C, injection volume 3 μL. MS data were acquired in positive/negative ESI modes using the following settings: Ion source: 425 °C, sheath/auxiliary gas 50/13 arb; Spray voltage: ±3.5 kV; Collision energy: 20–40–60 eV (stepped); Resolutions: Full MS 60,000, MS/MS 7500; Scan range: *m*/*z* 70–1050 (DDA mode). Quality control (QC) samples, prepared by pooling equal volumes of all extracts, were injected every 10 runs to monitor reproducibility.

Raw LC-MS data were preprocessed using Progenesis QI (Waters Corporation, Milford, MA, USA) to generate a three-dimensional matrix (CSV format) containing sample metadata, metabolite identities, retention times, *m*/*z*, and peak areas. The matrix was filtered to remove internal standards, false-positive peaks (noise, column bleed, derivatization artifacts), and redundant features via peak alignment. Metabolites were annotated using HMDB (https://www.hmdb.ca/), Metlin (https://metlin.scripps.edu/) and the Majorbio Database. Metabolic features detected in ≥80% of samples within any group were retained. Missing values (metabolites below LLOQ) were imputed with the minimum detectable value. Data were normalized to total ion current (sum normalization) and log10-transformed after excluding variables with RSD >30% in QC samples.

Principal component analysis (PCA) was performed on the samples (including QC samples) to provide an initial understanding of the overall metabolic differences between treatments and the sample variability within each group. During the PCA, the built-in statistical prcomp function of R software (version 4.2.1, https://www.r-project.org/) was used. The prcomp function parameter “scale” was set to “True” to normalize the data by unit variance scaling (UV). The Kyoto Encyclopedia of Genes and Genomes (KEGG) pathway enrichment analysis was conducted, and the Fisher was used to accurately determine the significance level of metabolite enrichment in each pathway, to identify metabolic and signal transduction pathways that have significant impacts. The metabolite enrichment in the KEGG pathway was significant when *p* was greater than 0.05. The information of DAMs was mapped to the KEGG database (https://www.kegg.jp) to obtain the KEGG metabolic pathways to which they were significantly enriched.

### 4.5. Data Analysis

The DAMs were selected according to the VIP values of the metabolites in the PLSDA (Partial Least Squares Discriminant Analysis) (or the VIP values of the PLSDA if the OPLSDA was overfitted) and the fold change and *p*-value in the univariate analysis, and the volcano map was plotted. The default criteria for selecting DAMs included the following: (1) Fold change = 1 (the significant difference in the abundances of metabolites between CK and T1/T2 group were more than 1-fold). (2) VIP ≥ 1 (VIP value indicates the intensity of the difference between treatments of the metabolites in the classification and discrimination of samples of the treatments). Heat maps (Tables S1–S3) were drawn by R software (version 4.2.1).

## 5. Conclusions

In this study, amino acid metabolism, arginine biosynthesis, ABC transporters, biosynthesis of alkaloids derived from shikimate pathway, and glyoxylate and dicarboxylate metabolism were the main pathways enriched by differentially abundant metabolites of *T. tortuosa* under Cd stress. The changes in the relative abundance of differentially abundant metabolites of *T. tortuosa*, such as succinic acid, N-Formylkynurenine, L-Alanine, L-Glutamine, and L-Histidine, were the mechanisms of *T. tortuosa* to resist exogenous Cd stress. In addition, the Cd content and antioxidant enzyme (SOD, CAT, POD) activity of *T. tortuosa* were closely related to the abundances of fusaric acid, N-Formylkynurenine, and succinic acid, indicating that fusaric acid, N-Formylkynurenine, and succinic acid were important compounds of *T. tortuosa* to resist exogenous Cd stress. This study delineates the metabolic adaptations and molecular-defense-related networks in bryophytes under cadmium stress, providing mechanistic insights that advance the development of phytoremediation technologies.

## Data Availability

The datasets generated during and/or analyzed during the current study are available from the corresponding author on reasonable request.

## Supplementary Materials

The supporting information can be downloaded at https://www.mdpi.com/article/10.3390/ijms26072856/s1.
