# Supplementary material for: Metabolomic Analysis of the Responses of Bryophyte Tortella tortuosa (Hedw.) Limpr. to Cadmium (Cd) Stress
Source: Int J Mol Sci. 2025 Mar 21;26(7):2856. doi: 10.3390/ijms26072856 (PMC11989171; doi:10.3390/ijms26072856)
Supplement: Supplementary file 1 [file ijms-26-02856-s001.zip › ijms-3520983-supplementary.pdf]

Table S1. Relative abundance of phyllosphere bacterial under different treatments.

	CK	T1	T2
Acidobacteriota	2572.67 a	3435.00 a	986.33 a
Actinobacteriota	2078.33 a	1673.33 a	585.00 a
Armatimonadota	100.00 a	34.33 a	43.33 a
Bacteroidota	1696.00 a	1415.67 a	1022.33 a
Bdellovibrionota	1090.33 a	710.00 b	133.00 c
Chloroflexi	1625.67 a	2203.00 a	127.00 a
Cyanobacteria	22200.67 a	23756.67 a	32656.67 a
Firmicutes	881.00 a	238.00 a	132.67 a
Gemmatimonadota	229.00 a	326.00 a	45.00 a
Myxococcota	440.00 a	561.67 a	199.67 a
Patescibacteria	101.00 a	79.67 a	99.00 a
Planctomycetota	818.67 a	354.33 ab	198.33 b
Proteobacteria	13983.33 a	15932.33 a	13785.67 a
Verrucomicrobiota	1968.00 a	559.00 b	489.33 b

Different lowercase letters indicate significant differences between groups ($p < 0.05$) determined by one-way ANOVA followed by Tukey's HSD post hoc test.

Table S2. Physiological characteristics and Cd absorption of *Tortella tortuosa* (Hedw.) Limpr. under different treatments.

	Chl a (mg/g)	Chl b (mg/g)	POD ($U\cdot mg^{-1}$(protein))	CAT ($U\cdot mg^{-1}$(protein))	MDA ($\mu mol\cdot mg^{-1}$(protein))	SOD ($U\cdot mg^{-1}$(protein))	Cd ($mg\cdot kg^{-1}$)
CK	3.62 a	1.81 a	52.08 a	5.84 a	10.84 c	13.69 a	0.34 c
T1	2.67 b	1.06 b	29.34 b	3.48 b	14.45 b	5.13 c	0.76 b
T2	2.06 c	0.71 c	17.78 c	2.21 b	15.86 a	11.72 b	1.89 a

Different lowercase letters indicate significant differences between groups ($p < 0.05$) determined by one-way ANOVA followed by Tukey's HSD post hoc test.

Table S3. Metabolites and main pathways of *Tortella tortuosa* (Hedw.) Limpr. under different treatments.

Metabolite	KEGG Pathway	P_value	CK	T2	T1
Uridine	ABC transporters	0.03804	5.659	5.472	5.503
Trehalose	ABC transporters	0.00311	7.163	7.464	7.149
Trans-Cinnamic acid	Amino acid metabolism	0.01238	6.146	6.51	6.117
Sucrose	ABC transporters	0.00311	7.822	8.04	7.748
Succinic Acid	Amino acid metabolism	0.00391	5.746	5.637	5.592
Shikimic acid 3-phosphate	Amino acid metabolism	0.03804	4.524	3.711	4.588
Serotonin	Amino acid metabolism	0.00091	3.83	5.486	4.377
Saccharopine	Amino acid metabolism	0.00234	3.727	4.621	3.885
Pipecolic Acid	Amino acid metabolis	0.00417	5.094	5.739	5.262
N-formylkynurenine	ABC transporters	0.03804	5.189	5.129	5.459
N-Acetyl-L-Glutamic Acid	Amino acid metabolism	0.00304	4.386	4.75	4.463

N6-(L-1,3-Dicarboxypropyl)-L-lysine	ABC transporters;Amino acid metabolism	0.00432	4.163	4.887	3.956
N2-Acetylornithine	Amino acid metabolism	0.00271	5.978	6.644	6.388
N2-Acetyl-L-ornithine	Amino acid metabolism	0.00271	5.438	6.114	5.856
Maleic Acid	Amino acid metabolism	0.00389	4.826	5.387	4.801
L-Tryptophan	Amino acid metabolism	0.00323	2.396	5.108	2.72
L-Threonine	ABC transporters;Amino acid metabolism	0.00341	5.55	5.979	5.555
L-Serine	ABC transporters;Amino acid metabolism	0.03192	5.475	5.74	5.485
L-Phenylalanine	ABC transporters;Amino acid metabolism	0.00363	4.365	4.758	4.308
LL- Orthophosphate c Acid	Amino acid metabolism	0.00251	4.408	5.35	4.8
L-Histidine	ABC transporters	0.00146	5.325	5.96	5.527
L-Glutamine	Amino acid metabolism	0.00091	5.723	6.553	5.974
L-Glutamic Acid	ABC transporters;Amino acid metabolism	0.04641	6.106	6.219	5.973
L-Glutamate	ABC transporters;Amino acid metabolism	0.02873	5.998	6.142	5.855
N-Formylkynurenine	Amino acid metabolism	0.00127	3.019	4.906	3.609
L-Alanine	ABC transporters;Amino acid metabolism	0.00255	5.272	5.339	5.119
L-2,4-diaminobutyric acid	Amino acid metabolism	0.00294	2.865	3.994	3.057
Kynurenine	Amino acid metabolism	0.00168	3.945	4.597	4.038
Indole	Amino acid metabolism	0.00186	6.188	6.272	6.213
Imidazoleacetic acid riboside	Amino acid metabolism	0.00234	3.085	4.499	3.466
Glyceric acid	Amino acid metabolism	0.01999	3.509	3.142	4.144
Diaminopimelic acid	Amino acid metabolism	0.03872	4.683	4.553	4.468
Deoxyguanosine	ABC transporters	0.00341	5.138	4.441	4.491
Citrulline	Amino acid metabolism	0.00168	3.664	5.325	3.836
ARGININOSUCCINATE	Amino acid metabolism	0.00141	4.589	5.421	5.032
Allysine	Amino acid metabolism	0.03192	4.774	4.654	4.603
Allophanic acid	Amino acid metabolism	0.01475	3.55	2.576	2.921
5-Hydroxylysine	Amino acid metabolism	0.02145	3.709	4.747	3.663
5-Hydroxy-L-tryptophan	Amino acid metabolism	0.00076	4.606	6.004	4.931
4-Aminobutyraldehyde	Amino acid metabolism	0.00136	4.517	5.061	4.686
2-Oxoarginine	Amino acid metabolism	0.00214	4.515	5.635	4.914
2-Hydroxycinnamic acid	Amino acid metabolism	0.00255	5.696	6.248	5.765
2-Aminomuconic acid semialdehyde	Amino acid metabolism	0.01886	5.824	5.898	5.814
2-Aminobenzoic Acid	Amino acid metabolism	0.00311	4.792	4.878	4.843
(S)-beta-Aminoisobutyric acid	Amino acid metabolism	0.00658	6.229	5.912	6.157
(S)-5-Amino-3-oxohexanoate	Amino acid metabolism	0.0394	4.762	5.092	4.78
(S)-3-Hydroxyisobutyric acid	Amino acid metabolism	0.03137	5.211	4.992	5.237
(E)-indol-3-ylacetaldoxime	Amino acid metabolism	0.00146	4.123	5.014	4.319
